# Genome flexibility in *Neisseria meningitidis*

**DOI:** 10.1016/j.vaccine.2009.04.064

**Published:** 2009-06-24

**Authors:** Christoph Schoen, Hervé Tettelin, Julian Parkhill, Matthias Frosch

**Affiliations:** a Institut für Hygiene und Mikrobiologie, der Universität Würzburg, Josef-Schneider-Strasse 2, Bau E1, 97877 Würzburg, Germany; b Institute for Genome Sciences, Department of Microbiology and Immunology, University of Maryland Biopark, 801 West Baltimore Street, Room 629, Baltimore, MD 21201, USA; c Wellcome Trust Sanger Institute, Hinxton, Cambridge CB10 1SA, UK

**Keywords:** Genomic flexibility, Meningococcus, Adaptation

## Abstract

*Neisseria meningitidis* usually lives as a commensal bacterium in the upper airways of humans. However, occasionally some strains can also cause life-threatening diseases such as sepsis and bacterial meningitis. Comparative genomics demonstrates that only very subtle genetic differences between carriage and disease strains might be responsible for the observed virulence differences and that *N. meningitidis* is, evolutionarily, a very recent species. Comparative genome sequencing also revealed a panoply of genetic mechanisms underlying its enormous genomic flexibility which also might affect the virulence of particular strains. From these studies, *N. meningitidis* emerges as a paradigm for organisms that use genome variability as an adaptation to changing and thus challenging environments.

## 1 Introduction

The first experimental investigations into the genome structure of *Neisseria meningitidis* began in the 1990s using pulsed field gel electrophoresis [1,2] and suggested a remarkable structural variability of the meningococcal chromosome [3]. For example, closely related strains such as those belonging to the electrophoretic type (ET)-5 or the ET-15 clonal complexes showed detectable differences in their macrorestriction patterns [4,5]. Perhaps even more surprising was the finding that within the course of a single infection the invading strain can undergo large genomic rearrangements such as the deletion of a 40 kb fragment [6]. These structural analyses were later complemented by genome-wide gene content comparisons, using representational difference analysis [7], of strains belonging to, amongst others, the ET-37 complex [8] and the multilocus enzyme electrophoresis lineage III [9], which also indicated a marked variability in gene content within these subgroups.

The advent of bacterial genome sequencing, beginning with that of *Haemophilus influenzae,* published in 1995 [10], sparked similar efforts in meningococcal research and transformed the entire field. The genome sequences of a serogroup A [11] and a serogroup B [12] strain were completed in the year 2000 and opened new avenues for basic as well as applied research. For example, the genome sequence of strain MC58 provided the starting point for the identification of novel target antigens for a serogroup B vaccine using “reverse vaccinolgy” [13] and served as a template for the computational reconstruction of the entire metabolism of *N. meningitidis*[14]. The complete genome sequences also enabled the design of whole-genome macro- and microarrays to analyse the gene content of larger collections of meningococcal strains by comparative genome hybridisation (mCGH) as well as the genome-wide investigation of the transcriptional responses to different experimental stimuli (reviewed in [15]).

Recently, two serogroup C strains [16,17] as well as three strains from healthy carriers [18] have been sequenced, bringing to seven the number of sequenced meningococcal genomes currently available for comparative analyses. Here, we provide a short description of the overall properties of the meningococcal genomes, highlighting their peculiar plasticity, and discuss recent analyses of the genomic differences between carriage and disease isolates.

## 2 The genomes of *N. meningitidis*: an overview

Currently, the genomes of four disease and three carriage strains have been sequenced with five genomes being completely sequenced and two genomes for which only draft sequences have been obtained (Table 1).

**Fig. 1:**
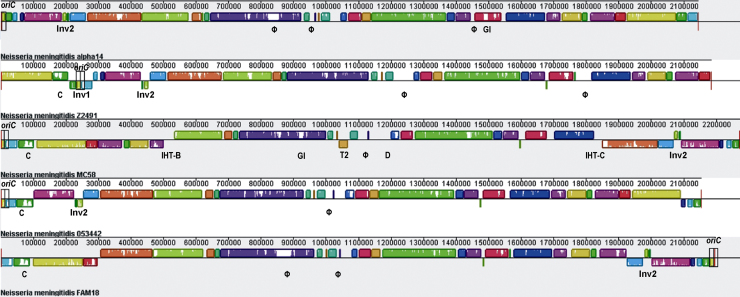
Annotated multiple whole-genome alignment using Mauve [71]. For each genome, the order of locally collinear blocks (LCBs) is given as a series of coloured blocks with the putative origin of replication designated *oriC* being indicated by a black rectangle. LCBs identically present in the four genomes are given in the same colours and horizontally flipped LCBs identify chromosomal inversions with respect to the genome of α14-like the inversion designated Inv1 in the genome of Z2491. Gaps or white spaces in the LCB order image indicate regions not (identically) present in all four genomes such as different prophages (Φ), genomic islands (GI), islands of horizontal transfer (IHT), or a region duplicated only in strain MC58 (D). In addition, the 20 kb region that is inverted in the four disease isolates (Inv) and the position of the capsule gene locus (C) are also shown.

Each sequenced genome consists of a single circular chromosome and the average genome size for all seven genomes is 2.193 (±0.056) Mb with an average G + C content of 51.63 (± 0.25) %. The common backbone of the five completely sequenced meningococcal genomes comprising the carriage strain α14 and the four disease isolates 053442, FAM18, MC58 and Z2491, respectively, is about 1.806 Mb in size and thus accounts for approximately 82% of the genome. However, the collinearity of the five completely sequenced genomes is broken by several segments of strain-specific regions and several inversions and translocations (Fig. 1). Of note, the average genome size of the carriage strains is not significantly different from the average genome size of the disease strains ((2.201 ± 0.73) Mb *vs*. (2.201 ± 0.051) Mb, *p* = 0.4, Wilcoxon rank sum test). This finding indicates that the evolution of virulence in *N. meningitidis* seems not to be the result of evolutionary processes leading to host restriction which in other bacteria, such as *Bordetella* spp. [19], has been shown to result in genome size reduction [20].

Accordingly, all other benchmark data such as G + C content, number of rRNA operons, tRNA genes or coding sequences (CDSs) are also quite similar for all strains irrespective of their origin (carriage or disease isolate), clonal complex or serogroup. In particular, all strains contain about 1971 (±78) CDSs with an average CDS length of 885 (±22) bp resulting in a coding density of 78.7 (±2.4) %. Besides these CDSs the genome of *N. meningitidis* also harbours a substantial number of pseudogenes. In particular, in a recent comprehensive analysis of pseudogenes in 64 prokaryote genomes, including 38 genomes from pathogenic species, the two *N. meningitidis* genomes investigated had the second and third highest proportion of pseudogenes, respectively [21].

## 3 Repetitive DNA and genomic plasticity

One of the most striking characteristics of the meningococcal genomes is the abundance and diversity of repetitive DNA that contributes to both genome fluidity and physical variability. For example, in a recent comparison of 53 complete bacterial chromosomes for repeats, *N. meningitidis* was found to have the 4th highest number, with about 20% of its chromosome consisting of repeated sequence of all kinds [22]. By analysing the loss of gene order conservation in 126 bacterial genomes and deriving an intrinsic measure of genome stability, *N. meningitidis* was assigned rank 121, and thus had the 6th lowest genomic stability [23]. The repeated sequences belong to a large variety of different classes which will be described briefly (Table 1) [11,17].

### 3.1 Families of repeated DNA sequences

The most obvious example is the neisserial DNA uptake sequence (DUS) involved in the recognition and uptake of DNA from the environment. There are nearly 2000 copies of the 12-bp uptake sequence in each genome which occur either alone or in inverted repeats as part of a transcriptional terminator [24]. Together with the high natural competence of the *Neisseriae* this large number of DUS might facilitate the incorporation of foreign DNA bearing the appropriate DUS from lysed bacterial cells of the same or related species. Remarkably, the DUS were not equally distributed over the entire genome and there was a significantly higher density of DUS within genes involved in DNA repair, recombination, restriction–modification and replication [25], indicating that transformation in *Neisseria* allows them to counteract deleterious effects of genome instability in the core genome [26].

**Fig. 2:**
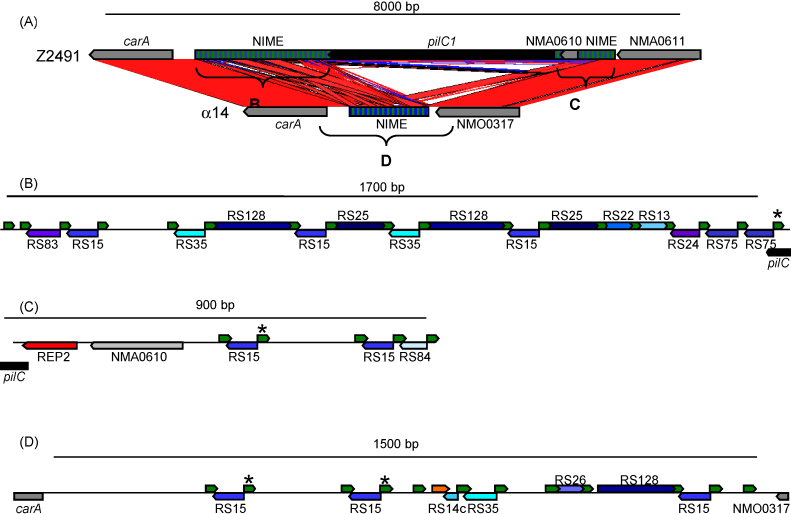
Region upstream of the *carA* gene coding for the small subunit of the carbamoylphosphate synthase in the genomes of *N. meningitidis* Z2491 and α14. (A) Comparison of the corresponding region in Z2491 and α14. The NIME repeat regions (hatched boxes) flanking the *pilC1* gene in Z2491 and the corresponding region in α14 are depicted in more detail in panels B, C, and D, respectively. Direct and inverted identical regions in both genomes are connected by red and blue lines and areas, respectively. (B) Repeat region between *carA* and *pilC1*, and (C) between *pilC1* and NMA0611, respectively, in Z2491. dRS3 repeats are depicted by green arrows and the names of the RS repeats (blue coloured arrows) are given. A red arrow indicates a REP2 repeat upstream of *pilC1*. (D) Corresponding repeat region in α14. Asterisks indicate identical dRS3 repeats that might constitute the integration or deletion site for *pilC1* and that are also target sites for the prophage Nf1 integrase. The orange arrow indicates a 28-bp region that is identical to the 3′ end of a silent *pilS* cassette (*pilS4* in Z2491 and *pilS2* in α14, respectively).

The second most abundant class of repeat sequences are the dRS3 elements which are a family of 20 bp repeats with conserved 6 bp terminal inverted repeats. They occur almost 700 times in the meningococcal genome and together with the families of 30–160 bp RS elements make up the so called “neisserial intergenic mosaic elements” (NIMEs) [11] (Fig. 2). These repeat elements in turn are often concentrated within intergenic repeat arrays of 200–2700 bp in size. Of note, it has recently been shown that the most abundant member of the dRS3 repeat family serves as target site for the chromosomal integration of a filamentous phage termed “neisserial filamentous phage” (Nf) 1 [27] or “meningococcal disease associated (MDA) island” [28]. Therefore, it was suggested that the phage integrase might catalyse also the recombination between different dRS3 elements resulting in permanent genomic changes in *N. meningitidis*, such as gene insertions and chromosomal rearrangements [18]. In addition, these repeat arrays might also serve to promote recombination with exogenously acquired DNA, increasing the rate of gene exchange at the adjacent loci. For example, a clear association was demonstrated between repeat arrays and genes encoding cell surface associated proteins where increased sequence variation may be an advantage in host interactions [17].

Also present in such repeat arrays are larger repeat units, which are also found in isolation, including the “Correia elements” (CEs) which represent about 2% of the *N. meningitidis* genome [29]. Correia elements are apparently mobile elements comparable to small insertion sequences (IS) of 100–155 bp in length with 26 bp inverted repeats and a TA target site duplication, but which, in contrast to conventional IS elements, do not encode a transposase [30,31]. Such non-autonomous mobile elements are common in eukaryotic genomes, where they are referred to as “miniature inverted repeat transposable elements” (MITES) [32]. Correia elements carry transcription initiation signals [33,34] as well as functional integration host factor binding sites [30,35], and hence may play a role in modulating the expression also of potential virulence genes. Computational genome comparisons suggest that CEs might be mobilisable possibly by the action of the IS*1106* transposase as suggested by their high sequence similarity to the N-terminus of the IS*1106* (IS*5* family) transposase gene. Like CEs, other repetitive extragenic palindromic sequences called REP2 were found to influence the expression of a set of virulence genes such as *pilC1* and *crgA,* which are necessary for the efficient interaction of *N. meningitidis* with host cells [36] (Fig. 2).

Another abundant but heterogeneous class of repeated sequences are so called simple sequence repeats or “contingency loci”, which comprise short tandem sequence repeats either within or 5′ to a coding region. The number of these tandemly repeated motifs can be modified during replication through slipped-strand mispairing and can consequently influence translation or transcription resulting either in a high frequency, reversible on–off switching of gene expression, or in an altered function and antigenicity of the encoded protein(s) [37]. Computational analyses identified over 80 potentially phase-variable genes in the meningococcal genome [17,38,39] with experimental evidence of phase variation for 15 genes (to date) encoding proteins which are mostly involved in the interaction with the host cells [40]. So far, compared to other bacterial species *N. meningitidis* seems to have the largest repertoire of phase-variable genes, accounting for almost 4% of all CDSs.

In most cases, phase-variable expression mediated by simple tandem repeats has been limited to the gene associated with the repeats. However, *N. meningitidis* also contains potentially phase-variable type III restriction–modification (RM) systems [41]. In some cases more extensive phase variation may be mediated by type III methyltransferases, as genes may come under the influence of the methyltransferases by point mutations generating a recognition site in a key position effecting transcriptional control of the gene. Therefore, phase-variable expression of the type III RM system may also influence the expression of multiple genes. Such a phase-variable regulon has been termed “phasevarion” [41].

Finally, the meningococcal genomes are also littered with insertion sequences (IS) and IS remnants, which constitute the largest size class of repeat elements. In particular, a comparative analysis of the distribution of transposases amongst 80 bacterial genomes revealed that *N. meningitidis* has the fourth highest number of IS elements with respect to genome size [42] with most of them belonging to the IS*5*, IS*30* and IS*NCY* families of IS elements, respectively. Of note, IS*1655* (IS*30* family) was shown to be restricted exclusively to *N. meningitidis* and absent from all other neisserial species, and its low sequence diversity was taken as evidence for a quite recent emergence of the species *N. meningitidis*[18].

This large variety of repeat sequences of all kinds provides the structural basis for the enormous genomic variability of *N. meningitidis*.

### 3.2 Intragenomic recombination and the generation of phenotypic diversity

*N. meningitidis* can be taken as a paradigm for genomic variability where the abundance of a diverse set of sequence repeats is exploited via recombination for the generation and maintenance of phenotypic diversity. Mechanisms for the generation of such diversity include phase variation based on slippage-like mechanisms (see above), C-terminal exchange in specific genes, and enhanced localised recombination and variation related to repeat arrays, as well as sequence conversion of expressed genes using information from silent loci.

As discussed above, genes where increased variation is beneficial, such as surface proteins involved in host cell interactions, are more likely to be associated with large repeat arrays. The resulting enhanced localised recombination and sequence variation can be seen for example in the *lbpAB* locus, encoding a lactoferrin-binding protein, the *tbpAB* locus, encoding a transferrin-binding protein, and the *porA* locus, encoding a major outer membrane protein, and the *pilC1*/*pilC2* loci [17] (Fig. 2).

The best described example for silent gene cassette-mediated sequence variation in genes coding for surface antigens is the pilin-encoding *pilE*/*S* system where the expressed pilin (PilE) can be altered by incorporation into the *pilE* gene of DNA from 5′-adjacent promoter-less *pilS* genes [43]. In *N. meningitidis* the silent *pilS* loci are embedded within NIME arrays (see above), and it is possible that specific dRS3-mediated recombination may contribute to generating silent variation within *pilS* sequences [17].

A different mechanism of variation appears to exist for several loci encoding putative haemaglutinins (*fhaB*) and adhesins (*mafB*). Downstream of these genes are what appear to be silent cassettes encoding alternative C termini for the encoded proteins. These cassettes contain short repeats that are identical to sequences only present within the upstream genes.

The *maf* loci are generally comprised of tandem *mafA* and *mafB* genes, both of which are thought to encode adhesins, followed by a number of putative silent cassettes. Each of the *N. meningitidis* genomes so far sequenced has three *maf* loci and, probably as result of recurrent intragenic recombination events with the silent cassettes downstream, there is considerable variation in the *mafB* genes, which would be expected to manifest as variations in adhesin structure at the cell surface [17].

Another example of such repeat-mediated rearrangements of the 3′-ends of genes encoding surface-exposed proteins are the homologues of the *Bordetella pertussis* filamentous haemagglutinin *fhaB*[11]. The *Neisseria meningitides fha* loci have similar structure to the *maf* loci with silent cassettes directly downstream of a gene encoding a large low complexity surface protein. The size of the repeats shared by the silent cassettes and the functional gene is comparable for both *fha* and *maf*. The *maf* and *fha* loci show considerable potential for generation of multiple versions of the expressed coding sequence and, together with surface structures such as pilus, capsule, and other surface proteins, are likely to be major contributors to cell surface diversity.

Finally, the *N. meningitidis* genomes contain three loci (RTX islands I–III) in which the *frpA*/*C* genes are located that code for iron-regulated type I secretion systems belonging to the RTX protein family [44] and which also seem to be involved in adhesion to epithelial cells [45]. Analogous to what was found for the *maf* and *fha* loci, the RTX islands also seem to include partial open reading frames that encode protein cassettes homologous to sequences within the RTX toxins. These RTX cassettes, in contrast to the *maf* and *fha* cassettes, encode protein sequences that differ in their N termini, whereas their C termini are homologous to regions internal in the toxins and conserved between them, suggesting that they can be exchanged to alter the N-terminal sequence of the secreted proteins [44].

In effect, recombination-driven genomic variation not only facilitates microbial evasion of immune responses, but the resulting polymorphisms influence all aspects of meningococcal biology [46].

### 3.3 Regions of horizontally transferred DNA

In addition to its flexible chromosome, *N. meningitidis* is able to modify its gene content via horizontal acquisition of DNA from the same or related species [47].

Regions of putatively horizontally transferred DNA in *N. meningitidis* can non-exclusively be divided into the so-called minimal mobile elements (MMEs) [48], islands of horizontally transferred DNA (IHTs) [12], canonical genomic islands (GIs) [49], and (defective) prophages.

A MME is a region between two conserved genes in which different whole-gene cassettes are found in different strains, and which are chromosomally incorporated solely through the action of homologous recombination [48]. Comparative analyses of the neisserial genome sequences revealed over 30 potential MME sites with many of them containing strain-specific genes that might have been acquired via horizontal gene transfer (HGT) even from other bacterial genera [50].

Still larger entities of laterally transferred DNA called genomic islands are frequently found in variable numbers in the genomes of many bacterial species. In general, they are associated with tRNA loci, are flanked by direct repeats, contain genes or pseudogenes coding for genetic mobility and are often characterised by atypical DNA composition [51]. Surprisingly, and in contrast to what has been found, for example, in the *Enterobacteriaceae*, in the *N. meningitidis* genomes sequenced so far only two regions were found to display the hallmark features of typical GIs. One is present in the genome of the strain MC58 and is a degraded form of a λ-like prophage present at the same locus in strains FAM18 and α14, respectively [49]. The second GI present in the genomes of strains α14 and α275 is also a degraded form of a prophage probably belonging to the family of the P4-like prophages.

In contrast, there are numerous regions in the meningococcal chromosome(s) called IHTs that do not necessarily show the organisational features of typical MMEs or GIs but nonetheless differ in their G + C content and codon usage [12]. For example, in serogroup A strain Z2491 they account for about 5% of the genome with identification of at least 60 coding regions, ranging in size from 224 bp to 11.3 kb [11]. Accordingly, there are a substantial number of genes also in the other meningococcal genomes that show a lower than average G + C content and that might therefore have been acquired via HGT (Table 1). Of note, most of them are also strain-specific (Fig. 1) and some of them code for proteins of paramount importance for neisserial–host cell interactions and pathogenicity, such as the synthesis of a polysaccharide capsule (see below).

In contrast to GIs or IHTs, so far no uniform criteria have been established for the identification of prophages in bacterial genome sequences [52]. In *N. meningitidis*, computational analyses revealed about 10 prophages, most of them being either defective mosaic relatives of the Mu-like group of prophages [11,12,17] or belonging to the family of the filamentous prophages called Nf [27]. The finding that at least the Mu-like prophage NeisMu1 in strain MC58 codes for membrane-associated antigenic proteins suggests that these proteins contribute to the variability in envelope structure and may thus influence virulence and pathogenicity [53]. In addition, the presence of the filamentous prophage Nf1 has been shown to be associated with certain hypervirulent meningococcal lineages, although it could not be shown that the phage itself codes for any virulence determinant [28]. However, as already mentioned the prophage Nf1 integrase might affect the expression of certain surface proteins involved in interaction with host cells by mediating genomic rearrangements at adjacent dRS3 repeat sequences [18].

## 4 Gene content and virulence evolution in *N. meningitidis*

Comparative genomic analyses revealed that a substantial proportion of the meningococcal chromosome might be imported via HGT which might therefore play a paramount role in shaping the meningococcal gene content. These analyses have also substantially enhanced our understanding of virulence evolution in *N. meningitidis* and in particular the evolution of the encapsulated strains.

### 4.1 The meningococcal pan-genome

**Fig. 3:**
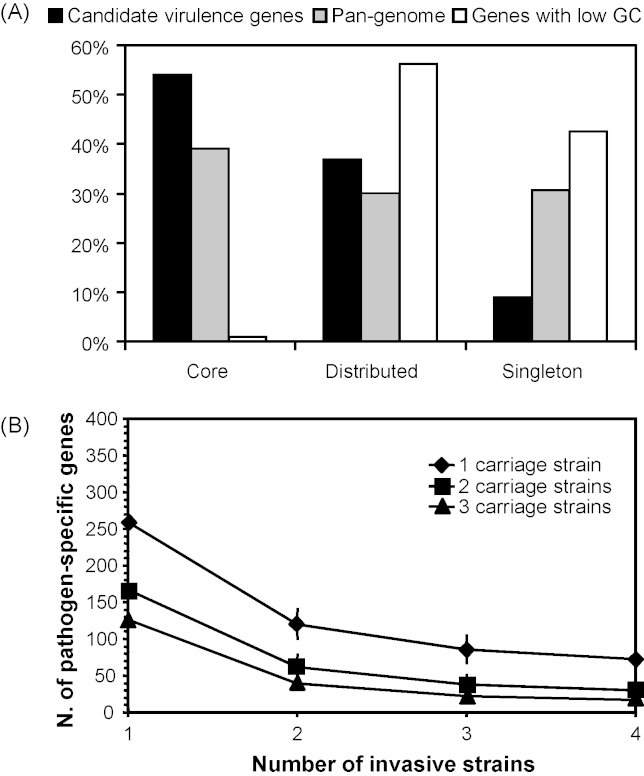
Gene content comparisons based on bi-directional best hits in all-against-all BLASTP comparisons [72] of the annotated coding sequences. (A) Distribution of different classes of genes amongst the different genomic compartments. Depicted is the partition of all genes from the species pan-genome (grey bar), genes having a low GC content and probably acquired via HGT (white bar), and the candidate virulence genes (black bar) into the core, dispensable and strain-specific genome. The core genome contains the genes that can be found in all strains, the dispensable genome contains the genes that are missing in at least one strain and the strain-specific genome comprises all the genes that can only be found in one strain. (B) For varying numbers of disease strains, the number of genes that are exclusively found in all disease strains and are absent in all carriage strains is plotted against the number of carriage strains compared.

The entire set of genes that can be found in the genomes of a species has been termed the “pan-genome”, which is composed of a “core genome” containing genes present in all strains, and a “dispensable genome” containing genes that are missing in at least one strain [54,55]. Based on bi-directional best hits in reciprocal TBLASTN comparisons of the annotated CDSs, the core genome of seven meningococcal strains might therefore contain about 1330 genes, whereas their pan-genome comprises about 3290 genes. Consequently, for the seven genomes compared, only 67% of the genes in each genome belong to the meningococcal core genome, which thus accounts for only about 40% of the pan-genome (Fig. 3A).

For an increasing number of genomes sequenced, the size of the core genome is expected to approach about 1300 genes (CI_0.95_ = [1257; 1327]), as indicated by non-linear regression analysis [54,55]. However, with increasing number of genomes the size of the pan-genome of *N. meningitidis* might follow a logarithmic trend, indicating that it grows albeit slowly but technically unbound, and that the number of new genes found in each genome might level off at around 38 new genes (CI_0.95_ = [27.7; 45.9]) per newly sequenced strain. Although more genomes might be required to reliably estimate the size of the meningococcal pan-genome, this result suggests that the meningococcal pan-genome might be open, and that capturing the entire gene pool accessible to *N. meningitidis* by whole-genome sequencing might thus be an elusive goal [18]. This result, however, might be less surprising given that *N. meningitidis* is naturally competent for the uptake of DNA, and might thus be able to acquire a large variety of DNA sequences from different sources via HGT.

### 4.2 The meningococcal pathogenome

With respect to the distribution of 134 recently compiled candidate virulence genes [56,57], gene content comparisons show that the presence or absence of these genes does not allow for a clear and consistent distinction between the four sequenced disease strains and the three sequenced carriage strains [18]. In particular, of the 19 candidate virulence genes that could not be found in at least one carriage strain, only three genes coding for FrpA/C-related RTX-family exoproteins were present in all four disease strains. However, all these genes code for proteins that have a number of highly similar paralogous genes in the genomes of the carriage strains. In addition, of the candidate virulence genes, 54% can be found in all seven strains, in contrast to only 40% of the entire pan-genome (see above) and 1% of genes having a lower than average G + C content (Fig. 3). In fact, candidate virulence genes are actually enriched for genes belonging to the core genome (OR = 1.83, CI_0.95_ = [1.277; 2.644], *p* < 0.001, Fisher's exact test) and should therefore more appropriately be considered as fitness genes being involved in, e.g., colonisation of the human nasopharynx and not as virulence factors for the invasion of host tissues.

In turn, by comparing the number of genes that are shared by all disease but absent from all carriage isolates it is evident that the size of the so-defined core pathogenome declines sharply with increasing number of genomes compared, and that the filamentous prophage Nf1 [27,28] might be the only genetic element that could be considered to be pathogen-specific by this analysis (Fig. 3B). However, as outlined already in the preceding sections, it was recently shown by mCGH that this genetic element is specific only for certain hypervirulent lineages but absent in others (reviewed in [15]), and that many other members of this family of phages exist in the sequenced genomes; therefore its contribution to meningococcal virulence awaits further experimental investigations.

### 4.3 Acquisition of the capsular locus via horizontal gene transfer

**Fig. 4:**
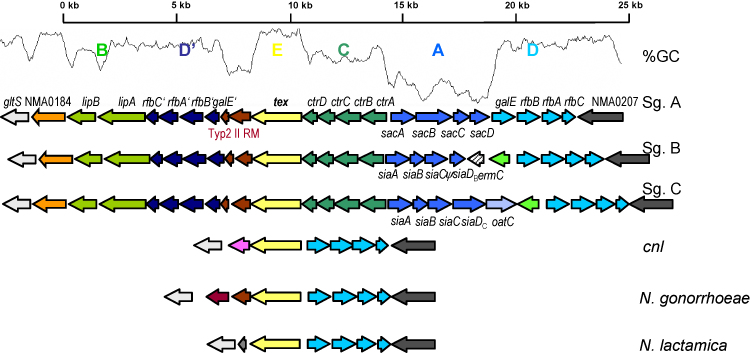
Capsule loci in different *Neisseria* species. The *cps* locus is flanked on either side by genes belonging to the neisserial core genome (coloured in white and dark grey, respectively). The five regions comprising the *cps* locus are depicted in different colours. Homologous genes are given identical colours according to the regions they belong to. In order to obtain an un-encapsulated and thus attenuated mutant strain of MC58, *ermC* coding for an erythromycin resistance gene has artificially been inserted into *siaD*_*B*_ and is thus not part of the *cps* locus. NMA0184 putatively coding for a TPR protein (COG0790) has not been described so far as part of region B. The genes coloured in dark green between regions A and D in MC58 and FAM18 and between regions B and D′ in α275, respectively, potentially code for proteins also involved in capsule synthesis. The genes coloured pink and light grey in strains α14 and *N. lactamica*, respectively, code for hypothetical proteins. In addition, the G + C plot for the capsule locus with Z2491 as reference is given.

The expression of a polysaccharide capsule is necessary but not sufficient to confer an invasive phenotype as revealed by epidemiological observations. However, it is the only factor that has so far been clearly associated with a pathogenic phenotype in *N. meningitidis*[58]. The *cps* locus required for the synthesis of the polysaccharide capsule consists of five regions, A to E [59] (Fig. 4). While regions E and D might belong to the neisserial core genome, as they can be found in many other *Neisseria* spp., regions A, B, and C, containing the genes required for capsule synthesis, modification and transport, respectively, can only be found in the encapsulated meningococcal strains. In line with an acquisition via HGT from other species, regions A and C reside on an island of horizontal transfer called IHT-A1, which has a lower G + C content when compared to the rest of the genome [12]. Also the *ctrABCD* genes of region C and the *lipAB* genes of region B are highly similar in sequence and operon organisation to the *hexABCD* (PMO0778-0781) and *phyAB* (PMO0772-0773) genes, respectively, in the *Pasteurella multocida* genome (GenBank AE004439). Therefore, the encapsulated and thus potentially pathogenic strains of *N. meningitidis* might have evolved from an un-encapsulated ancestor by horizontal acquisition of the *cps* locus from other bacteria residing in the human nasopharynx [18].

The large size of the *cps* locus (over 20 kb) and the conservation of region E (*tex*) in almost all neisserial species, which separates regions A and C from region B (and region D′), suggests that the acquisition of the capsule synthesis genes might have occurred in at least two steps, one possibly comprising regions A and C, and the other including region B. The conservation of region E and of the genes flanking regions A and C (*galE*) and region B (*gltS*), respectively, further suggest that these regions might have been imported in the form of two MMEs (see above) [50].

Accordingly, available biochemical data suggest that regions A and C may have been acquired after region B. If regions A and C would have been acquired earlier than region B by a putative progenitor of the encapsulated strains, this would imply that the export of the capsule polysaccharides via the region C-encoded CtrABCD transport system would also be possible without the action of the *lipAB* gene products. However, experimental inactivation of *lipA* and *lipB* resulted in intracellular inclusions of capsular polymers [60].

Of note, in all strains for which complete genome sequences are available, in addition to the acquisition of region B there is also a duplication of region D (resulting in region D′ next to region B), as well as a partially degraded type II RM system. It has been demonstrated that MMEs often harbour RM systems [50] and that the acquisition of type II RM systems can result in such coupled duplication/inversion rearrangements [61]. Closer inspection of the capsule locus finally suggests that a gene coding for a tetratrico peptide repeat (TPR) protein (COG0790) (NMA0184 in strain Z2491 taken as reference) has not been described so far as part of region B. Proteins containing TPRs are involved in a variety of biological processes such as – amongst others – transcriptional control or folding and transport of other proteins.

However, as it is hard to conceive what selective advantage the acquisition solely of region B might confer to *N. meningitdis*, an alternative hypothesis assumes the joint import of regions A, B and C on one fragment and a consecutive rearrangement event resulting in the separation of regions A and C from region B. In favour of the latter scenario is the observation of frequent genomic rearrangements at the *cps* locus [62] and that functionally coupled genes like those required for capsule synthesis are often found together on one mobile element [63]. Therefore, more experimental as well as sequence data from other encapsulated strains are needed to shed more light on this important event in the evolution of virulent meningococcal strains.

## 5 Genome-based reconstruction of meningococcal phylogeny

**Fig. 5:**
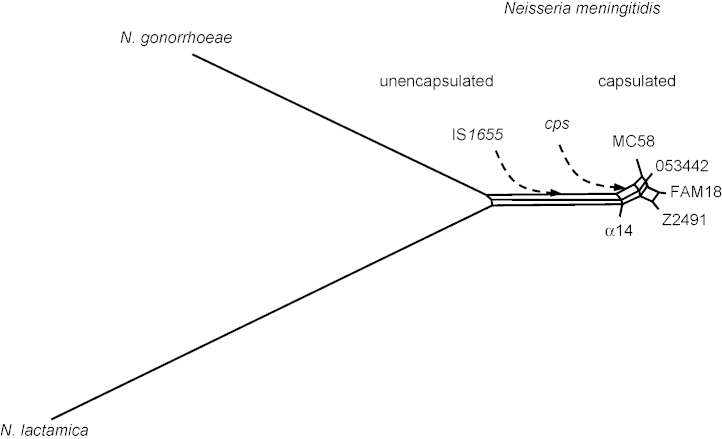
Hypothetical scenario of the evolution of encapsulated meningococcal strains from an unencapsulated common ancestor with *N. gonorrhoeae* and *N. lactamica* as suggested by whole-genome comparisons (neighbour net reconstruction [73] based on genome rearrangement distances [74]). A yet unencapsulated ancestor of *N. meningitidis* might first have acquired IS*1655* indicating the separation of *N. meningitidis* from other *Neisseria* species. At later time points the genes required for the production of a polysaccharide capsule (*cps*) were imported via HGT from other bacterial species, probably members of the *Pasteurellaceae* that also inhabit the nasopharynx of mammalian hosts.

In *Neisseria* spp., phylogenetic reconstructions based on single gene comparisons were shown to result in conflicting tree topologies due to intra- as well as interspecies recombination, as explained above [64]. In addition to recombination, comparisons based on small gene numbers can also generate inaccurate phylogenies because of sampling error or simply by the lack of sufficient amounts of data [65]. However, it has been argued that rare genomic changes such as sequence rearrangements offer alternative markers, where the linear information provided by, e.g*.*, concatenated sequence alignments as used in multilocus sequence alignments do not suffice [66]. This is because intrachromosomal rearrangements are not subjected to HGT events and therefore allow for a phylogenetic reconstruction even in the face of frequent inter-strain recombination [67]. As demonstrated in the preceding sections, *N. meningitidis* has a high number of repeated sequences of all kinds which can readily serve as target sites for homologous recombination events resulting in intrachromosomal rearrangements (Figs. 1 and 2). Based on rearrangements of locally colinear blocks of DNA in the 1.95 Mb common genomic backbone of the five completed meningococcal genomes and the full genome sequences of the two neisserial species *Neisseria gonorrhoeae* and *Neisseria lactamica*, a whole-genome-based reconstruction of neisserial phylogeny suggests that, amongst the five meningococcal strains compared, the unencapsulated strain α14 is the strain most similar to *N. gonorrhoeae* and *N. lactamica*. Therefore, α14 might be close to the branching point of *N. meningitidis* and *N. gonorrhoeae* from *N. lactamica*, and since *N. lactamica* and *N. gonorrhoeae* both lack a capsule this provides further evidence that *N. meningitidis* may have originated as an un-encapsulated species [18] (Fig. 5).

## 6 Summary and conclusion

*N. meningitidis* possesses a highly flexible and dynamic genome with respect to chromosome structure as well as to gene content. This flexibility might best be understood as adaptation to its life style as a commensal of the upper airways exclusively of humans where it must cope with local defence mechanisms and competing microbial species [46]. Comparative genome sequencing has provided evidence that *N. meningitidis* might in fact be an evolutionarily very recent species, with the encapsulated strains possibly having emerged within historical times [68,69]. This in turn would explain the highly similar genomic make-ups of disease and carriage isolates. Differences in the pathogenic potential between carriage and disease isolates might thus be influenced by small genetic differences in genes from the core genome, and it is therefore the “genetic personality” of a particular meningococcal strain, rather than the presence or absence of particular genes that might underlie their different pathogenic potentials. Future sequencing projects of hundreds of carriage and disease isolates with the latest high throughput sequencing technologies will shed further light on these subtle genomic differences and will provide further insights into adaptive mechanisms in this genetically highly versatile species.

## Figures and Tables

**Table 1:** Comparative overview of the sequenced meningococcal genomes.

Strain	Z2491	MC58	FAM18	053442	α14	α153	α275
Molecular typing							
Serogroup	A	B	C	C	cnl	29E	W-135
Sequence type	4	74	11	4821	53	60	22
Clonal complex	ST-4	ST-32	ST-11	ST-4821	ST-53	ST-60	ST-22
Frequency in carriers^a^ (%)	0.0	5.0	1.0	0.0	7.2	3.9	4.6
Frequency in cases^b^ (%)	0.0	23.8	18.1	0.0	0.0	2.7	0.4

General information							
No. of contigs (>2 kb)	1	1	1	1	1	87	133
Contigs/genome size (bp)	2184406	2272351	2194961	2153416	2145295	2134469	2266686
G + C content (%)	51.8	51.5	51.6	51.7	52.0	51.6	51.2
GenBank accession	AL157959	AE002098	AM421808	CP000381	AM889136	AM889137	AM889138

Functional RNAs							
Number of tRNAs	58	59	59	59	58	≥ 50	≥ 50
Number of rRNA operons	4	4	4	4	4	≥ 1	≥ 1

Selected repeats							
DUS^c^	1892	1910	1888	1858	1851	1831	1862
dRS3^d^	672	689	656	725	646	710	692
dRS3 (Nf1 subtype)^e^	303	316	283	327	269	322	307
CE^f^	270	261	n. d.	n. d.	269	n. d.	n. d.

Coding sequences							
Putative number	1993	2063	1975	2020	1987	≥1814	≥7
Average CDS length (bp)	902	871	918	853	884	899	870
COGs (%)	68	68	n. d.	n. d.	78	86	85
Coding area (%)	78.9	79.1	80.2	80.1	81.8	≥76.4	≥74.7
Putative pseudogenes	84	92	58	61	70	≥59	≥75
CDS with low G + C^g^	77	76	70	122	112	≥51	≥52
New genes^h^	57	46	9	12	58	≥2	≥42

a Frequency of clonal complex in 822 carrier isolates as given in [70].

b Frequency of clonal complexes in 525 disease isolates during the time period 2000–2002 in Germany, characterised by the European Meningococcal MLST Centre, Oxford, UK.

c The core DNA uptake sequence is GCCGTCTGAA.

d The dRS3 repeat sequence pattern is ATTCCC(N_8_)GGGAAT.

e The dRS3 repeat type targeted by the prophage Nf1 has the sequence ATTCCCRCCTRCGCGGRAAK [27].

f Correia repeat elements.

g Defined as genes with a G + C content less than the average G + C content of the CDSs in the respective genome minus two standard deviations.

h Defined as genes that have no hits in other genomes in TBLASTN comparisons.
